# Updates on Quantitative MRI of Diffuse Liver Disease: A Narrative Review

**DOI:** 10.1155/2022/1147111

**Published:** 2022-12-28

**Authors:** Marta Zerunian, Francesco Pucciarelli, Benedetta Masci, Francesco Siciliano, Michela Polici, Benedetta Bracci, Gisella Guido, Tiziano Polidori, Domenico De Santis, Andrea Laghi, Damiano Caruso

**Affiliations:** Radiology Unit, Department of Medical Surgical Sciences and Translational Medicine, Sapienza University of Rome-Sant'Andrea University Hospital, Via di Grottarossa, 1035-1039, 00189 Rome, Italy

## Abstract

Diffuse liver diseases are highly prevalent conditions around the world, including pathological liver changes that occur when hepatocytes are damaged and liver function declines, often leading to a chronic condition. In the last years, Magnetic Resonance Imaging (MRI) is reaching an important role in the study of diffuse liver diseases moving from qualitative to quantitative assessment of liver parenchyma. In fact, this can allow noninvasive accurate and standardized assessment of diffuse liver diseases and can represent a concrete alternative to biopsy which represents the current reference standard. MRI approach already tested for other pathologies include diffusion-weighted imaging (DWI) and radiomics, able to quantify different aspects of diffuse liver disease. New emerging MRI quantitative methods include MR elastography (MRE) for the quantification of the hepatic stiffness in cirrhotic patients, dedicated gradient multiecho sequences for the assessment of hepatic fat storage, and iron overload. Thus, the aim of this review is to give an overview of the technical principles and clinical application of new quantitative MRI techniques for the evaluation of diffuse liver disease.

## 1. Introduction

Diffuse liver diseases are highly prevalent worldwide conditions including pathological hepatic changes that occur when the hepatocytes are damaged and liver function decreases, and, most of the times, could lead to a chronic condition [1].

In clinical practice, the radiological evaluation is mostly qualitative; however, due to the rising prevalence of these diseases, there is a strong need of a highly accurate, standardized, and noninvasive quantitative assessment to manage diagnosis, therapy, and follow-up [2].

The actual reference standard for the diagnosis and quantification of diffuse liver diseases is percutaneous biopsy. However, this method is invasive, has low patient acceptance, suffers from low repeatability, and implies 3% risk of complications such as bleeding, pain, or infection with a mortality rate of 0.03% [3–5]. Other aspects that limit the clinical use of this procedure include the possibility of sampling errors due to the heterogeneous distribution of these diseases and intraoperator variability [6, 7]. For these reasons, noninvasive imaging methods have been progressively developed in the diagnosis and management of diffuse liver diseases.

Liver ultrasound (US) is the most common method used to assess fatty liver disease or fibrosis [4]; it is noninvasive, has wide availability in clinical practice, and is relatively inexpensive [2]. However, this technique has some limitations; firstly, it is operator dependent, which can lead to reproducibility issue; secondly, it does not visualize the entire liver (due to the interposing of ribs or abdominal gas); and thirdly, it could overestimate or misdiagnose steatosis with other diffuse liver diseases (e.g. inflammation or fibrosis) [2, 8]. Therefore, quantitative evaluation of diffuse liver disease with US is often imprecise, even though quantitative US based on the evaluation of attenuation coefficient and back scatter coefficient could grade steatosis or fibrosis better than conventional US [2]. Other US methods to quantify liver stiffness to evaluate diffuse liver diseases are based on elastography and the most common are transient elastography [9], shearwave elastography [8], and 2D static imaging ultrasound elastography [10]. Another important limitation of US technique is represented by the low sensitivity to detect mild steatosis; in fact, with histology as reference standard, US sensitivity and specificity to detect moderate to severe steatosis are 80–89% and 87–90%, respectively, that drops to 65% and 81%, considering all grades of steatosis [2]. This low sensitivity reflects the difficulty of an accurate steatosis grading by US, with consequent inappropriate therapy management or underestimation of future potential complications [11].

Computed Tomography (CT) is another imaging method used to assess diffuse liver disease considering the parenchymal attenuation on both unenhanced or enhanced images or by assessing at different morphological organ alterations [5]. Even if acquiring the images is relatively fast and not operator dependent, disadvantages include ionizing radiation exposure [12], decreased sensitivity in case of mild disease [2], and the possible influence of other substances such as iron in modifying the parenchymal attenuation.

Magnetic Resonance Imaging (MRI) is becoming the reference standard of noninvasive evaluation of diffuse liver diseases because it is the only technique that has been considered to have great accuracy for the diagnosis, staging, and follow up of these conditions [13–15]. One of the most innovative MRI applications is represented by MR elastography (MRE) that is able to identify fibrosis stage ≥2 (F2-4) and stage ≥3 (F2-4), with a combination of high sensitivity (91%) and specificity (96%) [16]. In fact, during the last years, there has been an intense technical development to the quantitative liver parenchyma assessment, both on unenhanced and contrast-enhanced imaging [17]. Among new MRI quantitative methods, T2/T2^∗^ sequence is applied for iron quantification [18], MRE for assessing liver stiffness by using mechanical shear waves that cause parenchymal vibrations [19], proton density fat fraction (PDFF) [20] that reflects parenchymal fat distribution by quantify chemical shift between fat and water, and diffusion-weighted imaging (DWI) with the possibility to quantify the molecule motion within a tissue [21]. In addition to all these MRI specific methods, radiomics represents another imaging technique that quantifies textural information of the spatial distribution of signal intensities and pixel interrelationship [22]. Although it is a technique not yet standardized among different imaging techniques, especially in MRI, it is a growing sector also in the study of diffuse liver diseases [23, 24].

Among all the imaging techniques available, MRI represents the most promising modality to quantitatively assess diffuse liver disease by using different methods and physics principle able to achieve a very high accuracy. Thus, the aim of this review is to give an overview of the technical principles and clinical application of new quantitative MRI techniques for the evaluation of diffuse liver disease. In addition, before the description of quantitative MRI tools, a brief recall about diffuse liver disease pathology and epidemiology will be included.

## 2. Diffuse Liver Diseases

Diffuse liver diseases occur when the hepatocytes and other cells composing liver parenchyma (i.e., Kuppfer cells, stellate cells, and bile ductular cells) are damaged by various aetiologies, with consequent alterations of the hepatic tissue that carries to the global decreases of liver function. The disease processes that diffusely affect the liver include excessive substances overload from exogenous intake or due to genetic alterations, immune or autoimmune disease, infections, vascular injuries, natural course of the majority of this pathological disorders leading to a chronic alteration of liver architecture with fibrotic changes, and cirrhotic alteration [1, 25].

The most common among diffuse liver diseases is nonalcoholic fatty liver disease (NAFLD); it is a metabolic disorder characterized by the accumulation of triglycerides, formed from the esterification of free fatty acids (FFAs) and glycerol within the hepatocyte and that owes its pathogenesis to the complex interaction between hormonal, nutritional, and genetic factors [26]; NAFLD is highly diffuse in the worldwide, with particular in the Western countries, as demonstrated by abdominal imaging studies where fatty liver is reported in at least 25% of American adults [27].

Another liver disease due to the accumulation of fat concerns alcohol-related liver disease (ALD) in which excessive alcohol consumption causes liver damage. The liver is the main organ responsible for the metabolism of ethanol and after excessive alcohol consumption, it suffers tissue damage due to oxidative stress, the accumulation of acetaldehyde and lipopolysaccharide (LPS). ALD is represented by a broad spectrum of diseases, including asymptomatic early ALD (fatty liver or steatosis), steatohepatitis, advanced ALD (alcoholic hepatitis, cirrhosis), and HCC attributable to alcohol consumption [28]. The progression of ALD is doses and duration-related but other aspects that concur to the ALD's development include genetic, epigenetic, and environmental factors [29]. About 20% of alcoholics and heavy drinkers develop fatty liver or steatosis and may progress to alcoholic hepatitis to cirrhosis, and it is estimated that 10% to 15% of alcoholics will develop cirrhosis [30].

Other diffuse liver diseases include intracellular overload of iron in hepatocytes, as it occurs in primary hemochromatosis, genetic condition that occurs approximately in 1/200-250 individuals, and haemolytic anaemia or in those who receive multiple blood transfusions [31].

A variety of diseases can result in abnormal accumulation of substances in the liver, in addition to fat and iron. Patients with hepatolenticular degeneration—Wilson's disease—have an abnormal accumulation of copper in the liver, as well as in the brain and cornea. Wilson's disease is an autosomal recessive genetic alteration with a frequency within the population of 1 in 30,000-40,000 [25].

Another systemic disease that causes diffuse liver disease is amyloidosis that is characterized by the extracellular deposition of amyloid protein in many organs. The progressive involvement of the organs leads to their malfunction and especially to death if there is an involvement of the heart and/or kidney. Liver and spleen are major sites of involvement. Amyloidosis is usually observed in a systemic form, but in 10-20% of cases, it can be localized. There are causes of secondary amyloidosis including multiple myeloma (10-15%), rheumatoid arthritis (20-25%), tuberculosis (50%), or familial Mediterranean fever (26-40%) [32]. The adjusted prevalence of amyloidosis increased significantly from 20.1 (15.5) cases per million in 2007 to 50.1 (40.5) cases per million in 2015, an annual percentage change of 12% (11.9%) [33].

A large family of liver storage diseases is represented by Glycogen Storage Diseases (GSDs) that inherited metabolic disorders of glycogen metabolism. The overall GSD incidence is estimated 1 case per 20000-43000 live births. There are over 12 types and they are classified based on the enzyme deficiency and the affected tissue including the liver, muscles, or both [34].

An important cause of diffuse liver diseases is also represented by viral hepatitis that show an acute onset but can subsequently evolve into chronic forms. Almost all cases of acute viral hepatitis are caused by one of the following viral agents: hepatitis A virus (HAV), hepatitis B virus (HBV), hepatitis C virus (HCV), the HBV-associated delta agent or hepatitis D virus (HDV), and hepatitis E virus (HEV). According to the World Health Organization (WHO), viral hepatitis still represents one of the main public health problems worldwide. In fact, WHO data report that in 2020, 325 million people in the world live with chronic hepatitis B or C infection and 1,300,000 people die every year from liver complications caused by infections [35]. The ability of acute forms to evolve in chronic disease varies from viral type; in fact, the forms that most commonly turn into chronic form are HCV and HDV, while HBV occasionally (1-10%) in adults; however, about 80-90% of children who contract the infection in the first year of life become chronic [25].

An important common factor that binds all these pathologies together is the development of cirrhosis that represents the end stage of mostly all the diseases cited. It is characterized by fibrosis and nodular hepatic regeneration of the liver parenchyma, which leads to the architectural subversion of the normal hepatic architecture replaced by connective tissue, leading to progressive hepatic failure [1]. Cirrhosis is a chronic condition which has a really high-social impact, considering that the incidence of new cases/year in Italy is around 30-60,000 new cases/year with a mortality rate that is luckily slowing down even if, in Europe, it is the fourth cause of death [36]. Prevalence is higher in males than in females, with important racial and socioeconomic differences and with greater prevalence in patients living below the poverty level [37, 38].

Among other causes of diffuse liver alterations is also important not to forget cardiovascular conditions and chronic cholestatic diseases. Cardiovascular alterations that can lead to cirrhosis are mainly related to right heart failure, constrictive pericarditis, Budd-Chiari syndrome [39], or veno-occlusive hepatic disease which is currently also called sinusoidal obstruction syndrome [40]. On the other hand, chronic cholestatic diseases comprehend different conditions, genetic or acquired, caused by autoimmune disorders, infections, toxic, or ischemic-related. Primary biliary cholangitis, most common in middle-aged females, and primary sclerosing cholangitis, more frequently in young to middle-aged males, are the most common primary cholangiopathies that could evolve to cirrhosis [41–43].

## 3. Diffusion-Weighted Imaging (DWI)

DWI is a MRI functional-imaging technique based on assessing the Brownian motion of water molecules in tissues, giving qualitative and quantitative information on the microarchitectural and microperfusional tissue structure, without the administration of a contrast agent [44].

In extracellular environments, water molecules have relatively free diffusion and intracellular molecules show restricted diffusion; water molecule diffusion is affected by different factors such as intracellular metabolites, extracellular structure, or cell's size [45]. Different tissues showed different diffusion properties and, among different structures, also liver parenchyma diffusion has its specific characteristics also influenced by age, sex, blood flow, the extracellular accumulation of matrix proteins, [44] or iron content [46]. The effects of diffusion are seen with MRI ultrafast pulse sequences or echo planar imaging (EPI) sequences, by adding a pair of bipolar gradients which rephases and phases the spins of the volume [47]; the phase dispersion is linked to the Brownian motion of the water molecules along the gradient direction. The amplitude and the diffusion of the gradients are represented by the *b* value, measured in s/mm^2^ [44]. There are two models to fit the information given by the DWI sequences: a monoexponential model, which requires a minimum of two *b* values, 0 and < or equal to 200 s/mm^2^, to obtain Apparent Diffusion Coefficient (ADC) [44] and a biexponential model which is used in a method known as Intravoxel Incoherent Motion (IVIM), which requires different *b* values to obtain several DWI images [47]. In the IVIM model, the capillary perfusion could be evaluated with low *b* values (<200 s/mm^2^) and the molecular perfusion with high *b* values (> or equal to 200 s/mm^2^), [21] from which three parameters are obtained: *D* or diffusion coefficient, *D*^∗^ perfusion or pseudodiffusion coefficient which reflects the microperfusion contamination of the diffusion's signal, and *f* or microperfusional fraction [44, 48]. ADC maps generally lowers when cell volume rises, as in cytotoxic cellular oedema, in acute infarcted tissues [45] or when cellular density increases, such as in cancer tissue.

As DWI is widely available, it has a relatively fast acquisition and does not need the administration of contrast medium, it has a potential filed of application in the quantitative assessment of diffuse liver diseases, for fibrosis and cirrhosis staging [49], as reported in Table 1. Some disadvantages are relative to the fact that DWI images are sensible to movement artefacts, often the ADC values are significantly influenced by cardiac movements on the left hepatic lobe and *b* value range is not standardized yet [47].

There are different studies in literature regarding the usage of DWI sequences in patients with hepatic steatosis or NAFLD. Guiu et al. [50], in a prospective study including 108 patients, observed that *D* value extracted from IVIM was lower in steatosic than in nonsteatosic liver (1.03 × 10^−3^ mm^2^/sec  ± 0.23 vs. 1.24 × 10^−3^ mm^2^/sec ± 0.15, respectively; *P* < 0.0001). Also *D*^∗^ was lower in steatosic livers compared with nonsteatosic liver (72.2 × 10^−3^ mm^2^/sec  ± 61.4 vs. 110.6 × 10^−3^ mm^2^/sec  ± 79; *P* = 0.0025), reflecting the perfusional reduction of liver tissue in steatosis. The *f* value was significantly higher in steatosic liver (33.8%  ± 9.4 vs. 26.9%  ± 8.8; *P* = 0.0003).

In a prospective observational study, Murphy et al. [51] analyzed the association between histologic alterations of the hepatic tissue and quantitative measures from DWI in 89 patients with NAFLD; the images obtained were analyzed with two different methods: conventional magnitude averaging (CMA) and the Beta^∗^LogNormal distribution (BLN). The results showed that ADC value decreased with inflammation and fibrosis, *D* decreased with steatosis and *f* decreased with inflammation NASH and fibrosis.

DWI has been evaluated also in infectious liver diseases, prevalently for assessing the chronic consequences such as advanced fibrosis or cirrhosis.

Lewin et al., [52] in a prospective study, compared DWI with noninvasive tests for the presence of liver fibrosis in 20 healthy volunteers and 54 chronic HCV patients, demonstrating that DWI is an excellent method for predicting severe or moderate fibrosis and cirrhosis. Patients with fibrosis between F2 and F4, showed hepatic ADC values lower than those with F0-F1 fibrosis. The AUCs for the discrimination of F3-F4 fibrosis patients from those with a fibrosis level of F0-F2 were 0.92 for DWI, 0.86-0.87 for other blood tests, 0.92 for FibroScan, and 0.79 for FibroTest. Sensitivity was 87%, specificity of 87%, PPV of 72%, and NPV of 94% for DWI.

Several studies have observed that both *D*^∗^ value and ADC significantly decreases in cirrhotic livers or in advanced stages of fibrosis; Taouli et al. [53] prospectively performed DWI on 23 patients with chronic hepatitis from various aetiologies (chronic HCV and HBV, nonalcoholic steatohepatitis, autoimmune hepatitis, and alcohol abuse) and on seven healthy volunteers and found a significantly lower value of hepatic ADC in stage 2 or greater fibrosis vs. stage 1 or less fibrosis and stage 3 or greater fibrosis vs. stage 2 or less fibrosis. The mean ADCs (×10^−3^ mm^2^/s) were 1.47 ± 0.11 versus 1.65 ± 0.10 for stage 2 or greater stage fibrosis versus stage 1 or less fibrosis (*P* < 0.001) and 1.44 ± 0.07 versus 1.66 ± 0.10 for stage 3 fibrosis or greater versus stage 2 or less fibrosis (*P* < 0.001). Also, hepatic ADC value was a significant predictor of stage 2 or greater fibrosis and stage 3 or greater fibrosis, with AUCs, sensitivity, and specificity, respectively, of 0.896 and 0.896, 83.3% and 88.9%, 83.3% and 80.0%.

Yoon et al. [54] in a retrospective study on 55 patients, showed a better diagnostic performance for evaluating hepatic fibrosis of IVIM DWI-derived parameters compared to ADC; both parameters showed significant correlation with fibrotic stages, being significantly higher in F0 to F1 than in F4 fibrosis stages (*P* < 0.05), but *D* showed significantly better diagnostic performance than ADC for diagnosing >F2 fibrosis (*P* = 0.009), with *D* decreasing along with the progression of the fibrosis (*P* < 0.05). Hu et al. [55], in a murine model, obtained the same results, as IVIM-derived parameters showed significant and better correlation with fibrosis stages than ADC parameter (AUC = 0.821 − 1.000 vs. 0.753-0.918).

DWI has also been used to evaluate liver iron overload in several studies; Akpinar et al. [56] observed a lower of ADC value as liver iron concentration severity increased in patients with *β*-thalassemia major, showing that DWI could be a predictor of iron overload with high sensitivity and specificity (AUC > 0.9).

Moreover, DWI has been used to evaluate different liver features in primary sclerosing cholangitis (PSC), in autoimmune hepatitis and in autoimmune sclerosing cholangitis [57]. A single-aetiology study performed by Kovač et al. [58] regarding PSC showed that the mean ADC values were significantly different between stages I and III (*P* = 0.01), stages I and IV (*P* = 0.001), and stages II-IV (*P* = 0.001), while ADC values at stages I and II, stages II and III, and stages III and IV were not as different.

Up to now, there is no literature regarding DWI in single-aetiology diffuse liver diseases such as amyloidosis, glycogenosis, or Wilson disease and this research field hopefully will expand in the future.

## 4. Fat Fraction and T2^∗^

For the quantification of liver fat and iron, biopsy continues to play an important role. Regarding fat accumulation, common MRI techniques are limited by T1 distortion, T2^∗^ decay, and interference effects of the proton multifrequency signal in the fat and eddy currents and may not be accurate enough in this way to quantify liver fat noninvasively [59].

There are resonance techniques, such as magnetic resonance spectroscopy (MRS) and magnetic resonance-fat fraction of the estimated proton density (MRI-PDFF), which are proving effective in quantifying these substances in a noninvasive way.

MRS is an imaging technique used in MRI to noninvasively evaluate the biochemistry and molecular composition of a tissue, using the signals given by the hydrogen protons to determine the concentration of the metabolites of interest within a tissue and has emerged as an accurate technique for quantifying liver fat [60]. Its limitations are due to the fact that MRS measures a small volume of sampled tissue and in this way is technically difficult to perform and is widely used for research with limited clinical availability and application in routine clinical practice [61].

New and state-of-the-art advanced MRI techniques are capable of eliminating all these observed biases through MRI-PDFF, a novel biomarker that has indicated a solid correlation and equivalence with MRS [62].

**Figure 1:**
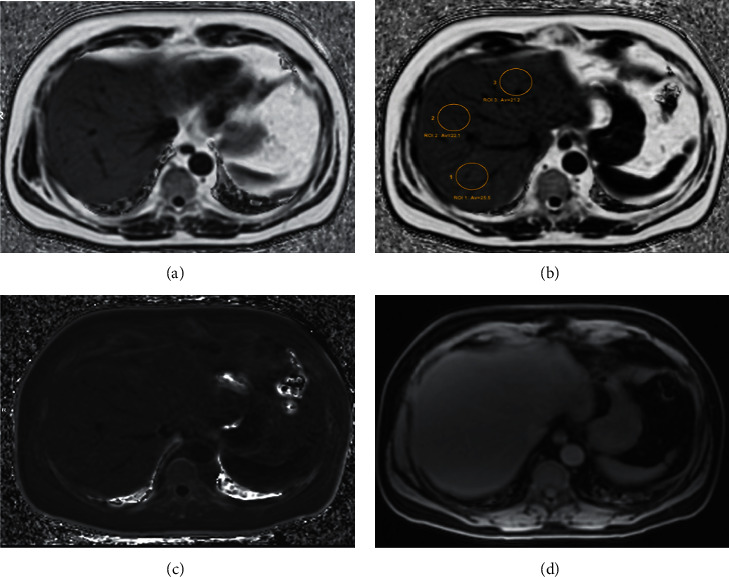
Fat fraction MRI images of a 65-year old male patient with NAFLD. (a) MRI-PDFF: 3D Ax IDEAL-IQ image. (b) MRI-PDFF: 3D Ax IDEAL-IQ image with ROIs placed showing high values of fat deposition than normal liver (>5%). (c) R2^∗^ 3D Ax IDEAL-IQ image representing the T2^∗^ correction needed to estimate MRI-PDFF. (d) Water 3D Ax IDEAL-IQ image as expression of different echo times used for the IDEAL-IQ sequence acquisition. MRI-PDFF: magnetic resonance-fat fraction of the estimated proton density; echo asymmetry and least squares estimation (IDEAL-IQ); NAFLD: nonalcoholic fatty liver disease; ROIs: regions of interest.

PDFF is an MRI technique that calculates the fat fraction at proton density and represents the proportion of a tissue composed of fat (Figure 1). It provides a quantitative assessment of hepatic fat throughout the liver by exploiting the resonance difference between the proton frequencies in water and fat by estimating the tissue fat fraction expressed in percentage. It works by separating the fat signal from the water signal, acquiring the gradient echo at specific echo times, and calculating the percentage of the combined signal that comes from the fat [63].

In addition to this, MRI-PDFF is able to give the possibility to map the amount of fat of the entire liver and can be applied on any clinical magnetic resonance platform, while MRS biochemically measures fat in small ROIs (2 × 2 × 2 cm^3^ voxel within the liver) [64].

Some drawbacks regarding the abovementioned techniques mainly concern the fact that, in patients with advanced liver fibrosis, the use of MRI-PDFF is limited by the severity of the fibrosis present [65] and to the fact that it is mandatory to determine MRI-PDFF thresholds that strongly correlate with histological grades of steatosis [66].

Noureddin et al. [64] conducted a three-way comparison of MRI-PDFF, MRS-PDFF, and the degree of steatosis histologically proven. They demonstrated in this longitudinal study that liver MRI-PDFF strongly correlated with liver fat content measured by MRS at both baseline and follow-up in 50 patients with biopsy-proven NAFLD. They also observed that changes in liver fat content measured by MRI-PDFF and MRS reflected changes in clinical/biochemical parameters such as weight and liver enzymes longitudinally and that liver fat content determined by MRI-PDF and MRS correlated with the degree of steatosis determined by histology. This study showed that hepatic MRI-PDFF is a reliable method to accurately quantify liver fat and a sensitive method to monitor changes in liver fat content in NAFLD patients. They also demonstrated that MRI-PDFF is highly correlated (Pearson correlation coefficient = 0.98) with MRS and exhibits excellent repeatability when the test is repeated on the same day (Pearson correlation coefficient = 0.99).

Tang et al. [67] assessed in a clinical prospective study the MRI-PDFF as biomarker of hepatic steatosis using histopathological analysis as a reference standard. They demonstrated that liver MRI-PDFF was systematically higher, with higher histologic steatosis grade (*P* < 0.001), and it was significantly correlated with histologic steatosis grade (*P* < 0.001).

Idilman et al. [68] evaluated the efficiency in the evaluation of hepatic fat using MRI-PDFF and MRS in patients with NAFLD and with liver biopsy as reference standard. For the quantification of hepatic steatosis, a close correlation was observed between the evaluation of steatosis in MRI-PDFF and in histology (*P* < 0.001) and between the steatosis detected by MRS liver and histology (*P* < 0.001) while no superiority between the two imaging methods was observed (*P* = 0.426). Although there is a strong correlation between the two MRI techniques, MRS measures a single-fat peak and therefore this could determine the underestimation of the fat fraction.

Beyond the quantification of intrahepatic fat, MRI is also a new great technique to quantify hepatic iron overload, particularly useful in hemochromatosis disease [69, 70].

MRE is a noninvasive test used to assess liver stiffness and fibrosis in chronic liver disease, which includes systemic iron overload. However, iron deposition by itself is associated with technical failure of MRE of the liver which necessitates the use of invasive liver biopsy as an alternative monitoring method for these patients. In that context, MRI has recently been used as an initial noninvasive diagnostic test to assess iron overload via the T2^∗^ parameter, which would be an exponential decay constant that can be calculated from a single-GRE sequence with multiple echo times. T2^∗^ sequences using gradient echoes and relatively long TE values are used to accentuate the effects of local magnetic homogeneity to aid in the detection of the overload of these paramagnetic substances [71]. T2^∗^ sequences demonstrated high sensitivity in detecting both hepatic and cardiac iron deposition, even in the early stages of the disease [72], and T2^∗^ values showed good correlation with the amount of hepatic iron accumulation compared with biopsy [73]. In addition to this, T2^∗^ allows the iron quantification from a large cross section of the liver compared to the biopsy that provides information only of small tissue sample.

Some limitations need to be addressed regarding T2^∗^ such as the evaluation of severe iron overload (>350 *μ*mol/g) that could be inaccurate and overestimated [74]; another aspect regards the need of a postprocessing algorithm that could not be universally available [74, 75].

**Figure 2:**
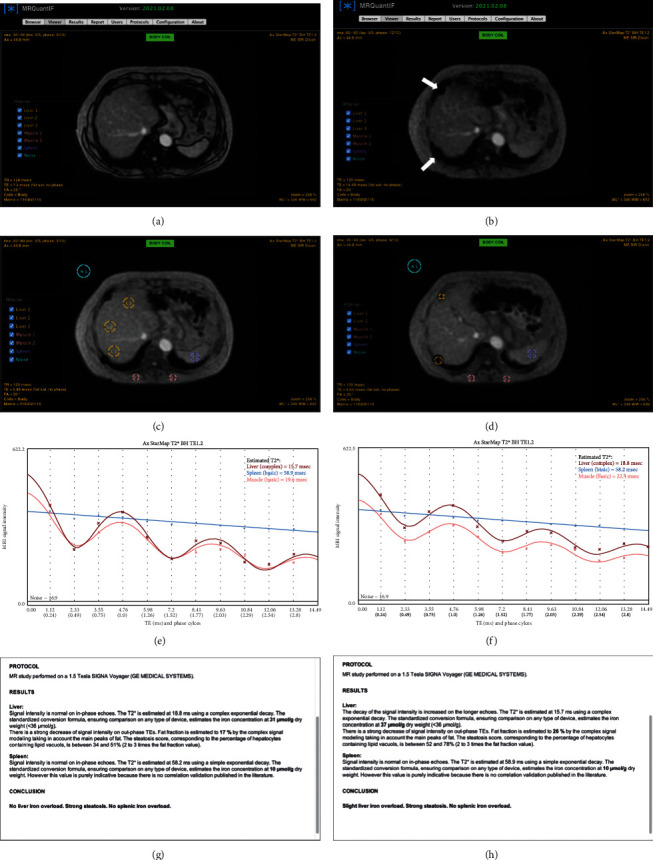
(a, b) StarMap images of a 65-year old male with heterozygous hemochromatosis and siderotic hepatic nodules (white arrows). (c, d) StarMap images postprocessing process with MRQuantiF software. ROIs are drawn on the liver parenchyma (c) and on hepatic nodules (d), on paravertebral muscles, spleen, and background noise for calculation. (e, f) Curves showing the estimated T2^∗^ timed of liver, hepatic nodules, spleen, and muscles. (g, h) represent the automatically reports generated by the software. ROIs: regions of interest.

Ghoz et al. [76] wanted to determine a cut-off value on the T2^∗^ reading at which the MRE would no longer provide accurate measurements of stiffness in iron-overloaded patients. To do this, they compared T2^∗^ values between patients with adequate MRE and those with nondiagnostic MRE and examined the ability of T2^∗^ to predict the probability of nondiagnostic elastography by estimating the AUC. Based on their results, T2^∗^ values can accurately predict which patients will have a nondiagnostic MRE exam, and specifically, they saw that a T2^∗^ of 20 ms or less (on a 1.5 T scanner) can indicate the possibility of a nondiagnostic MRE, values of 10, ≤8, and ≤6 ms seem to indicate a particularly high risk. To help the diffusion of these new tools, there are also some free online DICOM tools used to quantify iron and fat in the liver using a single-multiblind MRI sequence. Among these MRQuantif, [75, 77] provide a scientifically validated quantification of iron and liver fat from gradient echo sequences and is therefore able to evaluate the degree of steatosis and iron overload (Figure 2). It is therefore a simple software designed to assist radiologists in their clinical practice.

As we have seen, these methods provide quantitative and noninvasive evaluations in the diagnosis and follow-up in patients with hepatic steatosis or who have iron overload, such as in hemochromatosis. In this way, they could replace the use of biopsy which, as we have said, has many limitations and is not without risks.

## 5. MR Elastography (MRE)

MRE is an imaging technique used to quantitatively evaluate the mechanical characteristics of the liver based on the propagation of mechanical waves through the liver tissue [7]. MRE is among the most accurate noninvasive methods for detecting and staging liver fibrosis [9] and could probably replace liver biopsy in the future clinical setting [78, 79]. The shear wave generated during the examination has a propagating velocity in the tissue which is directly proportional to the level of stiffness [80] which is expressed in kilopascals (kPa) [81]; stiffness depends on many factors, such as the extracellular matrix of the organ or the internal pressure of the liver [82]. Normal stiffness liver values are considered lower than 2.5 kPa [7, 83–85].

**Figure 3:**
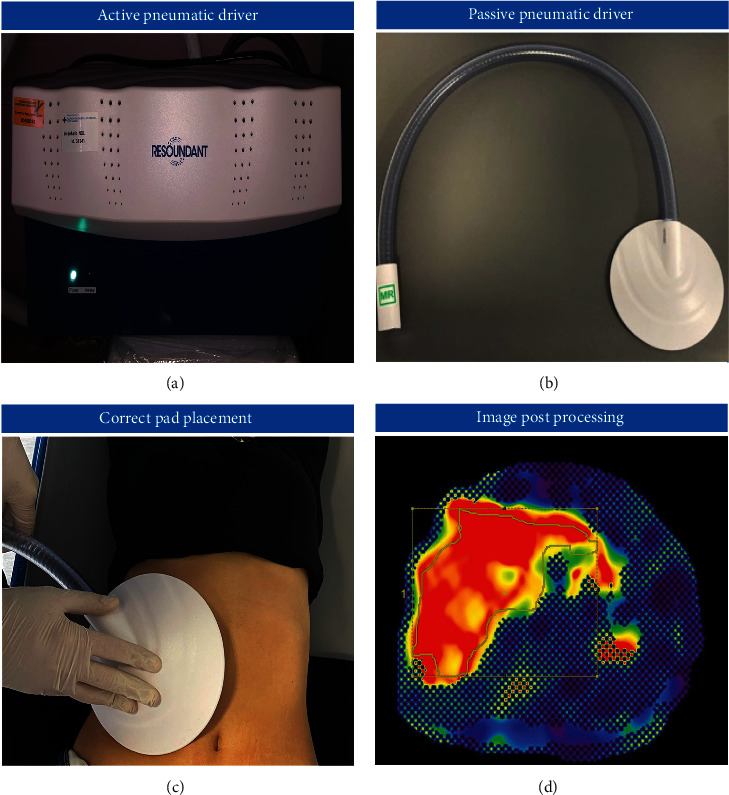
Technical aspects of performing MRE. (a) Active pneumatic driver. (b) Passive pneumatic driver (PAD). (c) The passive driver is correctly positioned over the right lower chest wall. (d) The image is postprocessed on dedicated work station. MRE: magnetic resonance elastography.

MRE can be performed both on 1.5 T or 3 T with a dedicated hardware to propagate mechanical waves and a specific software for processing images, as stiffness measurements depend on frequency and not on magnetic field strength [86]. To achieve its high reliability, this technique requires specific technical measures. Images for MRE are typically obtained with the patient in supine position, with a passive pneumatic driver, connected to an active pneumatic mechanical wave driver, that is positioned over the right lower chest wall in order to acquire the more hepatic tissue possible [3, 82], and held in place by an elastic strap (Figure 3). A continuous acoustic vibration is then generated and transmitted to the liver via the passive driver. The mechanical waves used in MRE are typically in the acoustic frequency range of 40-150 Hz and in literature, studies are prevalently performed with continuous vibrations at 50 Hz [87], 60 Hz [88], or 80 Hz [89]. In addition, patient preparation to the MRE includes fasting status, even if the technique does not required contrast medium injection, as postprandial status could increase hepatic stiffness in chronic liver diseases [90].

Axial images of the waves propagating in the liver are commonly acquired with gradient-recalled echo (GRE) pulse sequences with serial hold breaths of 15 seconds each [82, 84]; these sequences have special motion-encoding gradients synchronized to the frequency of the vibrations applied to the liver. An alternative to GRE sequences is spin echo planar imaging- (SE-EPI-) based MRE, which is faster than GRE and needs only one breath hold for multiple image acquisition [91]. A prospective study on 58 patients made by Serai et al. [92] demonstrated an optimal agreement in measuring liver stiffness between 2D GRE, with an interclass correlation coefficient (ICC) of 0.97; 95% confidence interval: 0.95, 0.98, and 2D SE-EPI, with an ICC, 0.98; and 95% confidence interval: 0.96 and 0.99 MRE. Wagner et al. [49] performed a prospective study involving 50 subjects in both 2D GRE and 2D SE-EPI MRE-imaging acquisition showing that the liver stiffness measurements did not differ significantly between the two sequences (3.75 ± 1.87 kPa vs. 3.55 ± 1.51 kPa, *P* = 0.062), but SE-EPI MRE sequence showed the advantage in terms of acquisition time and image quality in comparison with a GRE MRE sequence.

**Figure 4:**
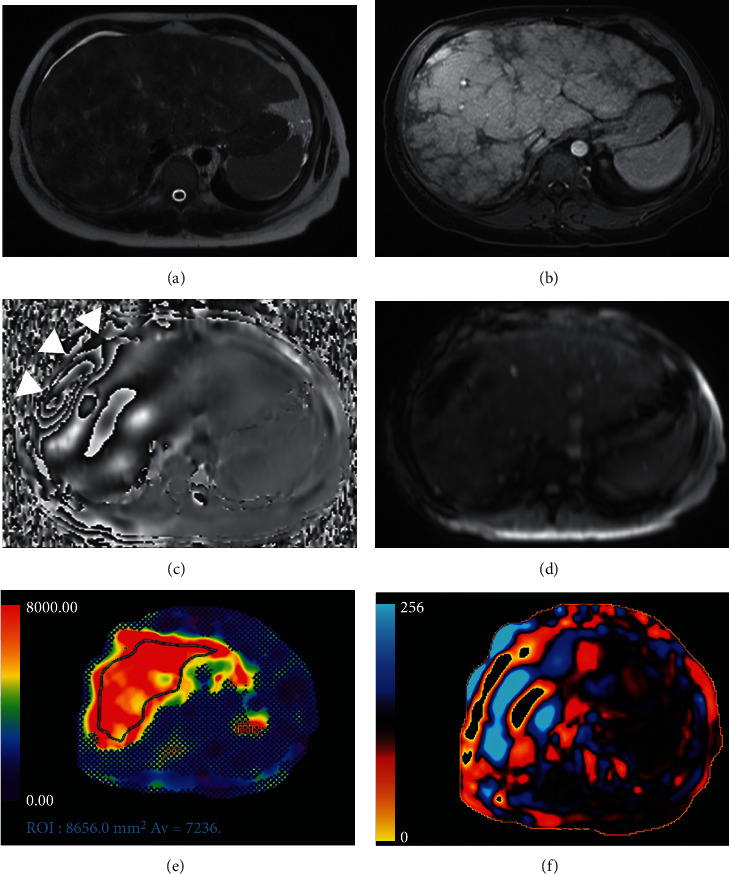
MRE images of a 38-year old female patient with NAFLD. (a) axial single-shot spin echo T2-weighted image shows the severe liver tissue alterations. (b) shows axial GRE T1-weighted fat-saturated image after contrast medium administration (gatoxetic acid), during hepatobiliary phase. (c) Axial GRE MRE image where it is possible to appreciate the propagation effect generate from the passive pneumatic driver (arrowheads). (d) Magnitude image. (e) Color elastogram with 95% confidence map. Free hand manual ROI is drawn on liver parenchyma on a large area of the liver that is not covered by the 95% confidence map shows an average stiffness value > 5 kPa, corresponding to a stage 4 fibrosis. (f) Axial wave image showing thick and irregular waves expression of hepatic fibrosis. MRE: magnetic resonance elastography; NAFLD: nonalcoholic fatty liver disease; GRE: gradient echo; ROI: region of interest.

As abovementioned, not only the image acquisition step is important to quantify liver stiffness. In fact, after image acquisition, MRE needs to be postprocessed with an inversion algorithm on a dedicated workstation; postprocessing includes the extraction from qualitative colour stiffness maps and grey scale stiffness maps. Then a quantitative liver stiffness measurements in kilopascals (kPa), range scale 0-8 kPa, a magnitude image which gives anatomic information and a phase contrast image which gives wave motion information, were generated [7, 84] (Figure 4). For stiffness quantification, free hand regions of interest (ROIs) are drawn on colour stiffness maps of each slice acquired (minimum number 4 slices), with magnitude image as optional anatomical reference, to obtain liver stiffness measurements [85]; Venkatesh et al. [84] suggest to exclude from ROI wave artefacts or large hepatic vessels, narrow segments of the liver, and the left liver lobe due to heart beat artefacts; also wave interference and low signal-to-noise ratio could lead to bias in the liver stiffness calculation. To consider MRE diagnostic, the total amount of pixels included in all ROIs should be at least 500 [93]. Guglielmo et al. [7] described the main causes of low quality elastograms, such as poor shear wave delivery to the liver, iron liver overload (3% of all examination in a cohort of 1377 patients [94]), or severe steatosis, motion artefacts, and interfering paramagnetic materials.

Among clinical applications, MRE has been mainly used to stage liver fibrosis, and a few data are present regarding other diffuse liver diseases.

In a retrospective study, Chen et al. [95] found out that in patients with NAFLD developing nonalcoholic steatohepatitis (NASH) without progression to fibrosis, liver stiffness was much higher than in those with simple steatosis (*P* = 0.028); MRE is considered a highly accurate method (area under the curve (AUC) = 0.93) for differentiating NASH from steatosis. In addition, liver stiffness was significantly correlated with inflammation grade (*P* = 0.0097) and fibrosis stage (*P* < 0.0001).

A prospective study performed by Loomba et al. [96] on 117 patients showed that MRE is accurate in predicting advanced fibrosis in NAFLD, with an AUC for discriminating stage 3-4 from stage 0 to 2 fibrosis of 0.924 (*P* < 0.0001).

MRE has been assessed also for studying infectious diseases of the liver. In a prospective study, Venkatesh et al. [97] measured the accuracy of MRE in detecting and staging liver fibrosis in patients with chronic HBV, in comparison with serum biomarkers; MRE resulted significantly more accurate than serum fibrosis biomarkers for detecting fibrosis (0.99 vs. 0.55-0.73) and cirrhosis (0.98 vs. 0.53-0.77). In addition, the study showed how MRE resulted accurate for significant fibrosis (F1-F2 METAVIR score) with a sensitivity of 97.4%, a specificity of 100%, a positive predictive value (PPV) of 100%, and negative-predicting values (NPVs) of 96%, while cirrhosis had a sensitivity of 100%, a specificity of 95.2%, a PPV of 91.3%, and a NPV of 100%. Moreover, Wang et al. [98] confirm the excellent performances of MRI in a retrospective study on autoimmune hepatitis, demonstrating a stronger correlation between liver stiffness with MRE and liver fibrosis stage compared to laboratory markers for chronic liver disease.

Yin et al. [80] also highlighted that with a shear stiffness cut-off value of 2.93 kPa, MRE could detect all grades of liver fibrosis with a sensitivity of 98% and a specificity of 99%; Receiving Operating Characteristic (ROC) curve also provided evidence that MRE can differentiate patients with mild fibrosis from those with grades 2-4 of fibrosis with a sensitivity of 86% and specificity of 85%. Similar results were found in a prospective study by Huwart et al. performed on 88 patients [99]. In addition to the pathologies described, some studies have evaluated specific liver stiffness measurements on single-specific aetiology such as alcoholic liver disease [100], in type 1 Gaucher disease [87], and amyloidosis [101], reporting some cut-off differences among them [85]; however, to simplify clinical routine, a more comprehensive cut-off has been proposed by experts applied on different aetiologies of chronic liver diseases [7, 85].

A specific limitation related to MRE is related to iron overload. In fact, in patients with high or moderate iron overload, MRI signal may be very low, with the impossibility of visualizing the waves with a GRE MRE sequence and consequent measurement failures [76]; Mariappan et al. [102] proposed alternative pulse sequences with shorter echo times even if severe iron overload still represents an important limitation to the technique. No studies on MRE in hemochromatosis have been published so far. As well as, no single-aetiologies studies for assessing liver stiffness measurements with MRE have been conducted yet in patients with Wilson disease, glycogenosis, primitive biliary cholangitis, or sclerosing primitive cholangitis. Future researches are needed to explore the wide spectrum of diffuse liver disease and MRE.

In conclusion, MRE has shown in recent literature a high PPV for ruling in moderate-significant fibrosis and a high NPV for ruling out cirrhosis and staging fibrosis. Future directions for MRE include the characterization of focal lesions and the differentiation between liver stiffness caused by fibrosis, oedema, inflammation, passive congestion, to assess precocious liver manifestations in context of alcohol consumption or in diabetes, to screen drugs mediated liver damages, or in monitoring the response in locoregional treatments for hepatocellular carcinoma (HCC) [103].

## 6. Radiomics

Radiomics is a quantitative tool applied to medical imaging, which aims to extract, from existing available data, ultrastructural pixel information regarding tissue characterization. The concept of radiomics has been applied more widely, but not exclusively, in the field of oncology [104, 105], and by mathematically extracting the spatial distribution of signal intensities and the interrelationships between pixels, it quantifies the texture information by using several analytical methods [22].

Thus, appreciable visual differences in image intensity, shape, or texture can be quantified by radiomics, overcoming the subjective nature of image interpretation and obtaining information not assessable by naked eye. Therefore, radiomics does not imply any automation of diagnostic processes but provides additional data to the existing ones, also creating an important quantitative tool to merge with the already existing biomarkers [22].

Radiomic analysis can be performed on different imaging modalities such as MRI, CT, and emission of positron tomography (PET); one of the first steps includes the two-dimensional or three-dimensional ROI segmentation (manual, semiautomatic, or automatic) to define the region in which the radiomic characteristics are calculated [106]. After segmentation, a dedicated software (open source or commercial) is necessary to process the images and to extract radiomic features [107, 108]. In particular, radiomic feature extraction refers to quantitative tissue descriptors expressed as grey level characteristics of the selected ROIs; radiomic features are extracted with different mathematical methods and are divided into different complexity order (first order, second order, or higher orders) to describe the pixel behaviour compared to the neighbouring pixels [22, 109]. The amount of the extracted features ranges from a few in first radiomic order to hundreds for higher orders; for that reason, it is necessary to select the most significant radiomic characteristics with specific technique (i.e., reproducibility analysis) and, only after the selection, used them for a multivariate model integrated with clinical and pathological data for the specific outcome (disease prediction, tissue characterization, staging, or survival) [110].

Regarding MRI in liver disease, there are many studies on radiomics focused on fibrosis characterization and staging and in differentiating focal liver lesions; unfortunately, the same cannot be said with regard to diffuse liver diseases such as storage pathologies, where radiomics applied on MRI still counts little literature compared to CT.

An example is provided by He et al. [23] that developed an automated model to classify hepatic stiffness, assessed with MRE which represented the reference standard, by using clinical and MRI radiomic features extracted from T2-weighted fast spin echo sequences. In this model, the combination of clinical and radiomic features showed the best performance (AUC = 0.84), compared to clinical (AUC = 0.77), or radiomic (AUC = 0.70) features alone to discriminate between fibrotic (>3 kPa) and nonfibrotic (<3 kPa) subgroups of patients with known or suspected liver disease. Using both clinical and radiomic features, the Supporting Vector Machine (SVM) model tested on 255 patients (training set) was able to correctly classify liver stiffness with an accuracy of 81.8%, a sensitivity of 72.2%, and a specificity of 87.0%; results were confirmed also in the validation set composed by 84 patients with accuracy, sensitivity, and specificity of 75.0%, 63.6%, and 82.4%, respectively (AUC = 0.80). Park et al. [111] demonstrated that a radiomic model-based derived from gadoxetic acid-enhanced hepatobiliary phase images allowed an accurate staging of liver fibrosis. The radiomic fibrosis index model created using data from the training cohort, including eight first-order histogram features and higher-order textural features including 24 gray-level cooccurrence matrix features and 11 gray-level run-length matrix features, allowed the stage of liver fibrosis in the test cohort with AUC of 0.90 and accuracy of 81%.

Ni et al. [112] optimized a model using MRI-unenhanced T1-weighted imaging to evaluate the staging of hepatic fibrosis in a rodent fibrosis model. To discriminate between the stages of fibrosis (F0-F4) grouped in different combinations (e.g., F0 vs. F1-2, F0 vs. F3-4, and F1 vs. F3-4), this study showed that least absolute shrinkage and selection operator-support vector machine (LASSO-SVM) and principal component analysis-support vector machine (PCA-SVM) showed an AUC ranging between 0.93 and 0.97.

Moreover, the study performed by Elkilany et al. [113] tested a radiomic model based to characterize and differentiate cirrhosis derived from different aetiologies (including viral hepatitis, cholestatic chirrosis, alcholic chirrosis, NASH, and autoimmune hepatisis) with inconclusive diagnosis from currently available noninvasive diagnostic tests. Three-hundred and six MRI examinations were included and 45 radiomic features were extracted from 2D ROI and volumetric 3D ROI on T1-weighted-enhanced sequences with gadoxetic acid at hepatobiliary phase. Results show that the five-fold cross-validated linear SVM allowed the aetiological classification of liver cirrhosis with AUC of 0.767-0.960, accuracies of 52.8-87.6%, and PPV of 0.377-0.883. During the study, they obtained the values of maximum accuracy (87.6% and 85.6%), sensitivity (97.6% and 95.6%), PPV (0.883 and 0.877), and the largest AUC (0.83 and 0.80) in 2D- and 3D-derived models, respectively, which differentiates cholestatic liver disease-induced cirrhosis from noncholestatic aetiologies.

As reported, MRI radiomics in all cases has provided promising results field of hepatic fibrosis staging. Hopefully, future research in the field of diffuse liver disease applied on MRI will achieve good performance to consider radiomics as additional noninvasive biomarker, even if studies in this field are still a few.

## 7. Future Directions and Conclusions

MRI is becoming a very important imaging technique in the evaluation of diffuse liver diseases, especially in the study of hepatic fibrosis, steatosis, and iron overload with different techniques such as MRE, DWI, MIR-PDFF derived from T1-weighted sequences, and T2^∗^. The main advantage is that it is a noninvasive method, it allows quantitative evaluation, it is non-operator-dependent, and it allows to evaluate the entire liver volume. Furthermore, radiomics applied to MRI provides a further step in the study of diffuse liver diseases with the possibility of retrospective analysis feasible on already acquired examinations.

Even though the imaging methods described to evaluate diffuse liver diseases are considered to be an alternative to liver biopsy, there are some limitations that needs to be addressed.

The main limitations of these new noninvasive biomarkers are related to the postprocessing software, not always available and that requires MRI machine, hardware, and work station updates, then that no studies in the literature regarding single-aetiologies diffuse liver diseases, and finally that clinicians are slightly sceptical in preferring these noninvasive techniques over the well-known liver biopsy.

Future directions of quantitative MRI of diffuse liver diseases include different imaging techniques such as MR fingerprinting applied to liver tissue to provide a faster quantitative assessment [114], deep-learning models to early detect liver fibrosis [115], and T1-mapping techniques to mainly evaluate the degree of hepatic inflammation, especially in NASH [6].

There are areas of recent active research regarding the use of hepatobiliary contrast agents to evaluate and quantify liver function, in terms of liver perfusion, vascular permeability, or expression of hepatocyte transporters, used to evaluate not only the degree of liver fibrosis but also the severity of cirrhosis [116, 117]. Different studies have considered the role of gadoxetic acid-enhanced MRI in distinguishing NAFLD from NASH and simple steatosis or staging fibrosis [118] with excellent results in terms of morphologic and quantitative evaluation of liver tissue. Up to now, the quantitative parameters considered in this imaging method remain limited by the long examination times requested, the complex mathematical models used, and the eventual presence of respiratory artifacts. Furthermore, no threshold values have been standardized to define normal and abnormal values [118]. Hopefully, future research will evaluate the functional and quantitative role of hepatobiliary contrast agents in diffuse liver diseases eventually with single-aetiology studies.

In conclusion, the main future goal would be to standardize the use of MRI in clinical routine for patients with diffuse liver disease as noninvasive, radiation free accurate method.

## Figures and Tables

**Table 1:** Magnetic resonance imaging studies on diffusion-weighted imaging for diffuse liver disease.

Study	*N* patients	Objective	Model performance	Reference standard	Nature of study
Guiu et al., Radiol. [50]	108	*D*, *D*^∗^, and *f* values derived from IVIM in steatosic livers vs. normal livers	*P* < 0.0001 *P* = 0.0025 *P* = 0.0003	Clinical assessment of type 2 diabetes	Monocentric Prospective
Murphy et al., J Magnetic Resonance Imaging [51]	89	Associations between histologic alterations and DWI in NAFLD	*P* < 0.05	Liver biopsy	Monocentric Prospective observational
Lewin et al., Hepatology [52]	74	DWI in predicting fibrosis or cirrhosis vs. healthy volunteers	*P* < 0.001	Liver biopsy	Monocentric Prospective
Taouli et al., AJR American Journal of Roentgenology [53]	30	ADC in quantification of liver fibrosis at different stages	*P* < 0.001 AUC = 0.89	Liver biopsy	Monocentric Prospective
Yoon et al., Journal of Computer Assisted Tomography[54]	55	IVIM parameters (*D*, *D*^∗^, and *f*) vs. ADC in hepatic fibrosis (F0-1 vs. F4)	*P* < 0.05 *P* < 0.009 *P* < 0.05	Liver biopsy	Monocentric Retrospective
Akpinar et al., Journal of Medical Imaging and Radiation Oncology [56]	55	ADC to assess iron overload in *β*-thalassemia major patients vs. iron overload MRI sequences	AUC > 0.9	Laboratory blood test for *β*-thalassemia major diagnosis	Monocentric Retrospective
Kovač et al., Acta Radiologica[58]	108	ADC in primary sclerosing cholangitis	*P* < 0.0001 *P* = 0.0025 *P* = 0.0003	MRI	Monocentric Prospective

D: diffusion coefficient; D^∗^: pseudodiffusion coefficient; f: microperfusional fraction; DWI: diffusion-weighted imaging; NAFLD: nonalcoholic fatty liver disease; ADC: apparent diffusion coefficient; AUC: area under the curve; IVIM: intravoxel incoherent motion.

## Data Availability

The data supporting reported results can be found from the corresponding author.
